# Structural and Functional Characterization of Anti-SARS-CoV-2 Spike Monoclonal Antibodies Produced via Bicistronic Expression in CHO Cells

**DOI:** 10.3390/antib14040086

**Published:** 2025-10-09

**Authors:** Federico Francisco Marsili, Fernanda Bittencourt de Aquino, Hiam Rodrigo da Silva Arruda, Mayra Amorim Marques, Katia Maria dos Santos Cabral, Marcius da Silva Almeida, Guilherme Augusto Piedade de Oliveira, Andrea Queiroz Maranhão, Renato Sampaio Carvalho, Leda dos Reis Castilho

**Affiliations:** 1 Cell Culture Engineering Laboratory, COPPE, Federal University of Rio de Janeiro (UFRJ), Rio de Janeiro 21941-596, RJ, Brazil; 2 Biochemistry Program, Chemistry Institute, Federal University of Rio de Janeiro (UFRJ), Rio de Janeiro 21941-909, RJ, Brazil; 3 Leopoldo de Meis Institute of Medical Biochemistry, Federal University of Rio de Janeiro (UFRJ), Rio de Janeiro 21941-902, RJ, Brazil; 4 Faculty of Pharmacy, Federal University of Rio de Janeiro (UFRJ), Rio de Janeiro 21941-902, RJ, Brazil; 5 Protein Advanced Biochemistry, National Center for Structural Biology and Bioimaging (CENABIO), Federal University of Rio de Janeiro (UFRJ), Rio de Janeiro 21941-902, RJ, Brazil; 6 Department of Cellular Biology, Institute of Biological Sciences, University of Brasilia (UNB), Brasilia 70910-900, DF, Brazil; 7 Laboratory of Pain and Inflammatory Pharmacology, Institute of Biomedical Sciences, Health Science Centre, Federal University of Rio de Janeiro (UFRJ), Rio de Janeiro 21941-577, RJ, Brazil

**Keywords:** monoclonal antibodies (mAbs), bicistronic expression vectors, IRES element, CHO cells, protein characterization

## Abstract

Background: Recombinant monoclonal antibodies (mAbs) represent the fastest-growing sector of the biopharmaceutical industry, with their efficient expression being a key technological factor for scalability. Objectives: In this study we compared the performance of two bicistronic vectors, which alternate the positions of the light and heavy chain coding genes, employing a wild-type Encephalomyocarditis virus (EMCV) IRES functional element to drive expression of the second gene. Methods: Using two neutralizing anti-SARS-CoV-2 IgG1 antibodies as model molecules, we conducted transient transfections in the commercially available ExpiCHO^TM^ platform. Following protein A affinity purification and quantification, vectors positioning the light chain as the first cistron consistently yielded higher expression levels than those with the heavy chain upstream. To confirm the quality attributes of the mAbs, we applied a comprehensive analytical workflow, including SDS-PAGE and Western blot for molecular mass and purity, circular dichroism for secondary structure, intrinsic tryptophan fluorescence for tertiary structure, and SEC-HPLC for quaternary structure and aggregate detection. Additionally, we assessed binding affinity to the target using spot blot and surface plasmon resonance, analyzed N-glycosylation profiles by HILIC-HPLC and mass spectrometry, and examined molecular structure by transmission electron microscopy. Results and Conclusions: Together, these results provide insight into the impact of gene positioning within bicistronic vectors on mAb expression efficiency and quality, supporting optimization strategies for scalable recombinant antibody production.

## 1. Introduction

IgG antibodies are complex heterotetrameric glycoproteins composed by two light chains (LC) and two heavy chains (HC), linked by disulfide bonds [1]. Their ability to selectively target different molecular epitopes has revolutionized modern medicine over the past two decades, as recombinant monoclonal antibodies (mAbs) have become the active ingredients of biological drugs, offering specific and effective treatments for several conditions or diseases [2].

An efficient expression of mAbs requires simultaneous synthesis of both polypeptide chains (LC and HC) in an adequate proportion. Different approaches have been described for mAb expression: (i) the co-transfection of two independent plasmids, each of them containing a transcription unit comprising either the LC or HC gene; (ii) the use of a single vector containing independent transcription units for the LC and HC coding genes, with expression of each gene under the control of independent promoters; (iii) the use of furin-2A-based polycistronic vectors, where 2A elements mediate a ribosome-skipping event and a furin cleavage site allows eliminating the additional amino acids of the 2A element that would otherwise remain attached to the upstream protein [3]; (iv) and the use of polycistronic vectors containing an internal ribosome entry site (IRES) [4,5]. An IRES is an RNA sequence most often found in the 5′ untranslated region (UTR) of viral mRNAs, which enable translation of the downstream mRNA sequence in a 5′cap-independent manner [6].

The co-transfection strategy (i) is widely used for mAb expression, but it might be strongly influenced by the integration site and efficiency, and by the number of the gene copies integrated into the cell host genome, which could lead to an unbalanced LC to HC expression and to a cell-to-cell expression variability. In the case of the multi-promoter approach (ii), there might be transcriptional interference between the two promoters, and a transcriptionally strong promoter could reduce or inhibit the activity of the more sensitive one [7,8,9,10]. Regarding the furin-2A (F2A) and IRES-based strategies (iii and iv), Ho et al. [3] have found higher titers for F2A-mediated tricistronic vectors, but stable expression based on this strategy led to unproperly processed species, incorrectly cleaved signal peptides and very high levels of aggregates, regardless of the relative position of the LC and HC genes within the tricistronic transcription unit. In contrast, the authors found that IRES-mediated tricistronic vectors resulted in correctly cleaved signal peptides and in mAbs with the expected size. When the LC gene was positioned upstream of the IRES element, high mAb expression levels and minimized product aggregation were found [3].

Among the IRES elements mostly used for multicistronic recombinant protein expression, the one originally identified by Duke et al. [11] and derived from the Encephalomyocarditis virus (EMCV) is one of the most widely used for recombinant mAb expression [3,7,12,13,14,15,16,17,18,19,20,21]. Previous works using IRES-based tricistronic vectors [13,14] have shown that high mAb expression levels were associated with an excess of LC, when its coding gene was positioned as the first cistron, compared to configurations where the HC gene was positioned as the first cistron. The third cistron contained the DHFR coding gene, which was preceded by a so-called attenuated version of the EMCV IRES element. Although the two IRES elements were described by these authors as “wild-type” (downstream of the first coding gene) and “attenuated” (downstream of the second coding gene) IRES elements, their sequence included an additional adenine residue at the oligo(A) viral regulatory sequence [22]. In the present study, we chose to use the native nucleotide sequence of the functional EMCV IRES element originally described by Duke et al. [11].

In addition to the careful selection of the expression strategies, the molecular complexity of mAbs demands detailed characterization of multiple physicochemical and biological properties, known as quality attributes (QAs), to ensure consistent production of safe and effective therapeutic antibody products [23]. This requires the application of a broad range of analytical techniques to evaluate parameters such as purity, intact recombinant protein molecular mass, primary structure, maintenance of higher order structures (secondary, tertiary, and quaternary), thermal stability, charge and size heterogeneity, post-translation modifications (e.g., glycosylation), biological and immunological properties, among others [24]. Certain QAs, including molecular mass, size, electrophoretic profiles, and antigen-binding affinity, are routinely assessed during early development stages, while others, such as impurity profiling and precise quantification of the mAb, become critical prior to clinical trials and eventual commercial release [25].

In this work, we compared the bicistronic expression and characterized several QAs of two neutralizing anti-SARS-CoV-2 spike mAbs, produced by transient transfection of ExpiCHO™ cells. Both mAbs were originally isolated from different COVID-19 convalescent patients by means of two different mAb discovery technologies [26,27], and were used as model mAbs for the investigation presented herein. We constructed bicistronic vectors alternating the position of the LC and HC coding genes in relation to a fully native EMCV IRES element and compared their expression levels. A comprehensive set of analytical techniques was used to investigate physicochemical and functional attributes of the mAbs. These efforts aim to contribute to the optimization of scalable recombinant mAb production techniques while ensuring consistent product quality.

## 2. Materials and Methods

### 2.1. Gene Synthesis and Vector Design

**Figure 1:**
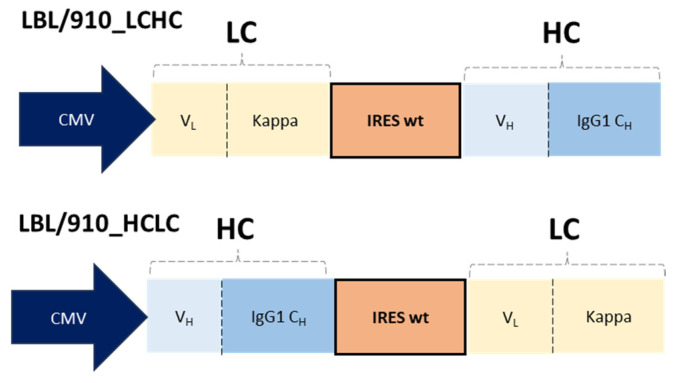
Schematic representation of the bicistronic expression cassettes bearing either the LC (LCHC) or the HC (HCLC) genes as the first cistron. A wild-type EMCV IRES element was used to enable bicistronic expression. In the case of mAb LBL-01, the human IgG1 C_H_ domain contains a LALA mutation [28], whereas for mAb 910-30 a human wild-type IgG1 C_H_ coding sequence was used. CMV: cytomegalovirus promoter; LC: light chain; HC: heavy chain; IRES: internal ribosome entry site; wt: wild-type; V_L_: light chain variable domain; V_H_: heavy chain variable domain; C_H_: heavy chain constant domain.

mAb 910-30 is an IgG1 that was discovered and first described by Banach et al. [26], whereas mAb LBL-01 (also an IgG1) was discovered and first described by Conde et al. [27]. The genes coding for the light and heavy chains were codon-optimized for expression in CHO cells and synthetized at Genscript (Piscataway, NJ, USA). Bicistronic vectors coding for each mAb, either containing the LC or the HC gene in the first cistron (Figure 1), were built using the pCI-neo vector (Promega, Madison, WI, USA) as backbone. A wild-type EMCV IRES element that had been described previously [13,20] was used to enable bicistronic expression.

### 2.2. Transient Expression in ExpiCHO^TM^ Cells

ExpiCHO™ cells (#A29133, ThermoFisher, Waltham, MA, USA) were used for mAb production by transient transfection. For routine maintenance of cells, the viable cell density (VCD) was adjusted every 2–4 days to 0.2–0.5 × 10^6^ cells/mL in 20–25 mL of ExpiCHO™ Expression Medium (ThermoFisher, Waltham, MA, USA), and cells were kept in vented Erlenmeyer flasks in a humidified incubator at 37 °C and 5% *v*/*v* CO_2_, shaken at 125 rpm on an orbital shaker with 25 mm stroke. For transient mAb expression, ExpiCHO™ cells were transfected according to the “standard protocol” for transient expression described in the manufacturer’s instructions. Briefly, cells were expanded and VCD was adjusted to 3–4 × 10^6^ cells/mL in ExpiCHO^TM^ Expression Medium one day before transfection. On the day of transfection (day 0), VCD was adjusted to 6 × 10^6^ cells/mL just before transfection, and cells were transfected using 1 μg of plasmid DNA and 3.2 μL of ExpiFectamine™ CHO reagent (ThermoFisher, Waltham, MA, USA) per mL of cell suspension. On day 1, 6 μL of the ExpiCHO™ Enhancer reagent and 0.24 mL of the ExpiCHO™ Feed (both from ThermoFisher, Waltham, MA, USA) were added per mL of cell suspension. These transient transfections were carried out in duplicate for 9–11 days post-transfection (dpt) at 20 mL working volume in 125 mL Erlenmeyer flasks (for both mAbs) or at 300 mL working volume in 1000 mL Erlenmeyer flasks (for mAb LBL-01 only) under the same conditions of temperature, CO_2_ atmosphere and agitation as used for routine cell maintenance. VCD and cell viability were determined by the trypan blue exclusion method using a Vi-Cell XR automatic cell counter (Beckman Coulter, Brea, CA, USA). Supernatant samples (3 μL) were analyzed by spot blot assay on a nitrocellulose membrane, using a goat anti-human H+L HRP-conjugated antibody (#A18805, Invitrogen, Waltham, MA, USA) at 1:10,000 dilution, followed by incubation with Clarity^TM^ Western ECL substrate (#1705061, Bio-Rad, Hercules, CA, USA) for chemiluminescent detection.

### 2.3. mAb Purification by Protein A Affinity Chromatography

The contents of each duplicate of shake flasks in both scales were harvested by centrifugation at 2000× *g*, pooled together and filtered using 0.45-μm PVDF membranes (Merck KGaA, Darmstadt, Germany). The purification runs were carried out using a 5 mL Eshmuno^®^ A Minichrome column (Merck KGaA, Darmstadt, Germany) in an ÄKTA Purifier system (Cytiva, Uppsala, Sweden) with online measurement of pH, conductivity and absorbance at 280 nm. The clarified supernatants (SNs) were injected at 5 mL/min, and the equilibration and washing steps were carried out using 0.1 M sodium phosphate buffer (pH 7.4), whereas elution buffer was 0.1 M sodium phosphate buffer/phosphoric acid (pH 3.0). Eluted fractions (5 mL each) were neutralized by adding 0.5 mL of 1.0 M Tris-HCl (pH 8.0). Protein concentration in the eluted fractions was measured at 280 nm in a NanoDrop 2000 spectrophotometer (ThermoFisher, Waltham, MA, USA). Fractions corresponding to the elution peak were pooled, concentrated approximately 2.6–4.0× using Amicon^®^ Ultra-15 centrifugal ultrafiltration devices (Merck KGaA, Darmstadt, Germany), filtered with 0.22-μm syringe devices (TPP, Trasadingen, Switzerland) and maintained at 4 °C until subsequent analyses.

### 2.4. SDS-PAGE and Western Blot Analysis

Samples were analyzed by sodium–dodecylsulfate–polyacrilamide gel electrophoresis (SDS-PAGE) and Western blot under non-reducing and reducing conditions, in 4-20% polyacrylamide Mini-PROTEAN^®^ TGX Precast Gels (#4561093, Bio-Rad, Hercules, CA, USA). Samples were mixed with 4× Laemmli sample buffer (#1610747, Bio-Rad, Hercules, CA, USA) and then incubated at 99 °C during 5 min and loaded onto the gels (4 μg of protein per well for SDS-PAGE and 2 μg per well for Western blot). The molecular mass marker used was Precision Dual Colour Plus Marker (#1610374, Bio-Rad, Hercules, CA, USA). When reducing conditions were used, β-mercaptoethanol (#1610710, Bio-Rad, Hercules, CA, USA) was added to the sample preparation to a final concentration of 0.355 M. Gels were run in Tris-glycine premixed buffer (#1610771, Bio-Rad, Hercules, CA, USA) at 150 V. For the SDS-PAGE gels, Coomassie blue staining was carried out using with the reagent Fast-blue Stain Reagent (Scienco Biotech, Lages, Brazil), according to manufacturer’s instructions. The recombinant mAbs were detected in the Western blot assay using a goat anti-human H+L HRP-conjugated antibody (#A18805, Invitrogen, Waltham, MA, USA,) at 1:10,000 dilution, followed by incubation with Clarity^TM^ Western ECL substrate (#1705061, Bio-Rad, Hercules, CA, USA) for chemiluminescent detection. In the gels and Western blots related to structure characterization (Section 3.2), the commercially available humanized anti-IL6 IgG1 for therapeutic use Actemra^®^ (tocilizumab, TCZ, manufactured by Roche, Basel, Switzerland) was used as an IgG1 control.

### 2.5. Evaluation of Secondary and Tertiary Structures

Secondary structure was investigated by circular dichroism (CD) spectroscopy in a J 1500 CD spectrophotometer (Jasco Inc., Easton, MD USA). Concentration of mAb samples was adjusted to 100 μg/mL in phosphate buffer, and spectra were collected in the far UV interval of 190–260 nm (bandwidth = 2 nm), using a 1 mm quartz cuvette. For tertiary structure assessment, tryptophan intrinsic fluorescence was measured using an ISS K2 steady-state Spectrofluorometer (ISS Inc., Champaign, IL, USA). Using the same sample concentration of 100 μg/mL, tryptophan fluorescence emission (selectively excited at 280 nm) was collected in the range of 300–400 nm in triplicates. Actemra^®^ (Roche, Basel, Switzerland) was also included as an IgG1 control.

### 2.6. Quaternary Structure and Heterogeneity Assessment by SEC-HPLC

Purified samples (300 μL, at a protein concentration of 0.025 mg/mL) were injected in a LC-20AT HPLC system (Shimadzu Corp., Kyoto, Japan), using a Superose-6 Increase 10/300 GL column (Cytiva, Uppsala, Sweden) and phosphate buffer as the mobile phase. Actemra^®^ (Roche, Basel, Switzerland) was included as an IgG1 control.

### 2.7. Transmission Electron Microscopy (TEM)

Grids were cleaned for 45 s at 25 mV using a GloCube^®^ Plus glow discharge system (Quorum Technologies, Laughton, UK). Samples were diluted 50-fold in ultra-pure water and immediately applied to the carbon side of the discharged 300 mesh copper grids (TedPella Inc., Redding, CT, USA). After 1 min of incubation, grids were gently dried with filter paper and stained thrice for 10 s with a freshly prepared 15 mg/mL uranyl formate solution (Electron Microscopy Sciences, Hatfield, PA USA). Negatively stained samples were imaged on a Tecnai microscope operated at 80 kV at the Advanced Microscopy Unit of the National Centre for Structural Biology and Bioimaging (CENABIO/UFRJ). A total of 10 micrographs were acquired using a CCD camera, resulting in the detection of 326 individual particles. The micrographs were processed using the single-particle analysis workflow in CryoSPARC [29]. Contrast transfer function (CTF) estimation was performed solely to verify the quality of the images. Particles were manually picked and extracted with a box size of 90 pixels. Initial 2D classification was performed to remove damaged or low-quality particles, followed by multiple rounds of 2D classification to identify well-defined particle classes. Selected 2D class averages were then used to generate a low-resolution ab initio 3D model. Homogeneous refinement was applied to the 3D reconstruction, and the final map was visualized from two distinct orientations.

### 2.8. N-Glycosylation Profile Evaluation by HILIC-HPLC and LC-MS

#### 2.8.1. High-Performance Hydrophilic Interaction Liquid Chromatography (HILIC-HPLC)

In order to obtain an overall view of the glycosylation pattern of the mAb molecules, a PNGase F kit (New England Biolabs, Ipswich, MA, USA) was used to digest purified mAb samples according to manufacturer’s instructions. After PNGase F digestion, samples were treated with cold ethanol to precipitate proteins, and the supernatant fraction containing the glycans was recovered, dried under vacuum and labelled with 2-aminobenzamide (2-AB). 2-AB excess was eliminated by preparative paper chromatography, whereby the glycans were recovered from the chromatography paper by elution with ultra-pure water. Samples were filtered and injected in a TSKGel^®^ Amide-80 column (4.6 mm × 25 cm, 5 μm) (Tosoh Biosciences, Tokyo, Japan) using an LC-20AT HPLC system (Shimadzu Corp., Kyoto, Japan) with the fluorescent detector set to 330 nm excitation and 420 nm emission. Conversion of the retention time (RT) of the peaks into glucose units (GU) was performed using an adjusted third-degree polynomial model, obtained from a reference run with a 2AB-labelled hydrolyzed dextran ladder prepared in-house from the commercial product Dextran Standard 5000 (#00269, Sigma-Aldrich, St. Louis, MO, USA).

#### 2.8.2. Hydrophilic Interaction Nano-Liquid Chromatography/Tandem Mass Spectrometry (LC-MS)

In order to enable exact identification of the glycans present in the mAb LBL-01, purified mAb samples were denatured at 100 °C for 10 min in 200 µL of denaturation buffer consisting of 40 mM DTT (#43819, Sigma-Aldrich, St Louis, MO, USA) and 0.5% *m*/*v* SDS (#L3771, Sigma-Aldrich, St Louis, MO, USA) in water. Following denaturation, samples were allowed to cool to room temperature and subsequently subjected to enzymatic deglycosylation with PNGase F. The reaction was carried out at 37 °C for 18 h in a final mixture containing 25 µL of 10% *v*/*v* NP-40, 25 µL of 500 mM sodium phosphate buffer (pH 7.4), and 700 mU of PNGase F (#F8435, Sigma-Aldrich, St Louis, MO, USA). After PNGase-F digestion, samples were treated with cold ethanol to precipitate proteins and then desalted. The glycans recovered in the supernatant were derivatized with procainamide, followed by a second desalting step and a drying phase (as described by Selman et al. [30]), with final solubilization in water/acetonitrile prior to the LC-MS analysis. Sample duplicates were filtered and injected in a TSKGel^®^ Amide-80 column (4.6 mm × 25 cm, 5 μm) (Tosoh Biosciences, Tokyo, Japan) using a Nexera X2 HPLC system (Shimadzu Corp., Kyoto, Japan), followed by analysis in an MS Maxis Impact system (Bruker, Billerica, MA, USA) fitted with an electrospray ionization source. The relative abundance of N-glycan species was calculated from the area under the peaks corresponding to their base-peak chromatograms (BPC), using the open software MZmine (version 2.41.1). These analyses were carried out at the multiuser Center for Biomolecule Mass Spectrometry (CEMBIO) of the Federal University of Rio de Janeiro (UFRJ).

### 2.9. Evaluation of Affinity to the Target by Spot Blot and Localized Surface Plasmon Resonance (LSPR)

The affinity of the recombinant mAbs to recombinant SARS-CoV-2 spike proteins of the D614G, delta, gamma, beta and omicron BA.2 variants (all of them produced by stably transfected HEK293 cells and purified at LECC/COPPE/UFRJ, Brazil) was first evaluated by a qualitative spot blot analysis. Briefly, different amounts (300, 200, 90, 30 and 3 ng) of purified spike proteins were pipetted onto a nitrocellulose membrane, incubated with each of the recombinant anti-SARS-CoV-2 mAbs (at protein concentration of 1 μg/mL), and then incubated with a goat anti-human H+L HRP-conjugated antibody (#A18805, Invitrogen, Waltham, MA, USA) at 1:10,000 dilution, followed by incubation with Clarity^TM^ Western ECL substrate (#1705061, Bio-Rad, Hercules, USA) for chemiluminescent detection. Localized surface plasmon resonance (LSPR) experiments were additionally carried out to quantitatively evaluate the affinity of both mAbs to D614G, delta, gamma and omicron BA.2 spike variants using an OpenSPR™ equipment (Nicoya Life Sciences, Kitchener, ON, Canada), a protein-A sensor kit (#SEN-AU-100-10-PROA-KIT, Nicoya LifeSciences, Kitchener, ON, Canada) and 0.1 M phosphate buffer (pH 7.4) as the mobile phase. Protein A coupling to the carboxyl sensor was performed following the manufacturer’s instructions: briefly, an initial surface conditioning was performed with 10 mM HCl, followed by surface activation (with the EDC and NHS reagents provided in the kit), protein A addition and a final blocking step with ethanolamine. Immediately thereafter, each anti-SARS-CoV-2 mAb was added to the protein A containing sensors at a concentration of 20 μg/mL. The D614G, delta, gamma and omicron BA.2 spike variants were then added at different concentrations (100, 50 and 25 nM) in duplicates. System regeneration was achieved with glycine solution (pH 2.0) after every experimental run. LSPR data was acquired and processed using the software GraphPad Prism 8.0.2 (GraphPad, San Diego, CA, USA). K_D_ values were determined using the BIAevaluation software (version 4.1), selecting a Langmuir model which minimizes both the associated chi-squared (χ^2^) and the standard error values.

### 2.10. Statistical Analysis

Significant differences between different experimental conditions were assessed using one-way ANOVA followed by Sidak’s multiple comparisons post hoc test, using the software GraphPad Prism 10.1.2 (GraphPad, San Diego, CA, USA).

## 3. Results and Discussion

### 3.1. Transient mAb Production in ExpiCHO™ Cells

**Figure 2:**
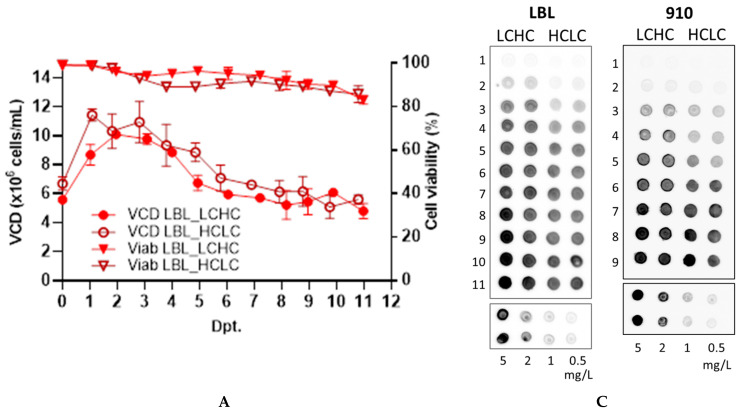

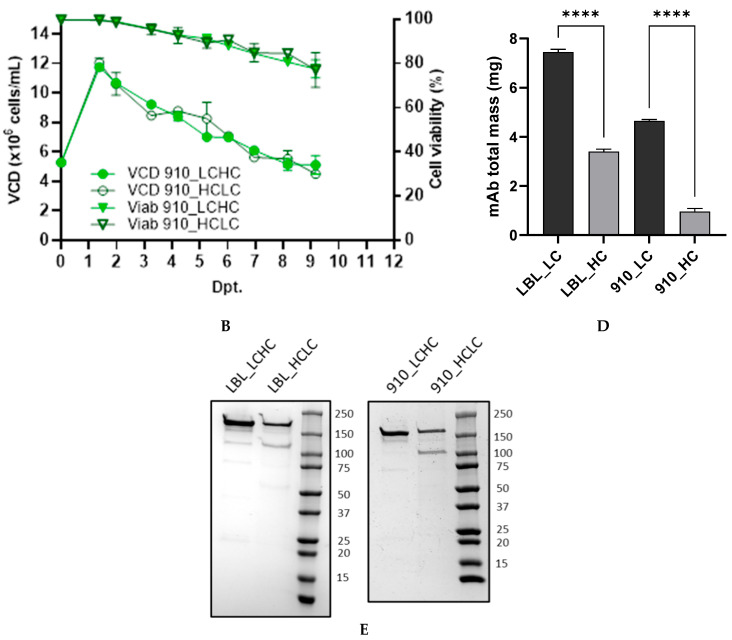
Viable cell density (VCD) and cell viability of transiently transfected ExpiCHO™ cells for expression of (**A**) mAb LBL-01 and (**B**) mAb 910-30. Both LCHC and HCLC bicistronic constructs were used to transfect the cells, according to the standard protocol described in the ExpiCHO™ expression kit. Results are shown as the average values of experimental duplicates. (**C**) mAb LBL-01 and mAb 910-30 secretion detected in cell culture supernatants by spot blot assay. The numbers at the left indicate the respective days post-transfection (dpt) of each sample. For mass yield comparison, a purified human IgG standard at varying concentrations (mg/L) was included as a reference. (**D**) The contents of each experimental duplicate were pooled together and filtered, yielding in all cases a volume of supernatant of 34 ± 1 mL, which was purified using protein A affinity chromatography. The recovered mass was calculated based on the protein concentration of the main fractions, determined by measuring absorbance at 280 nm. **** Indicates statistically significant difference (*p* < 0.0001) by one-way ANOVA test followed by Sidak’s multiple comparisons post hoc test (**E**) The purity of the pooled and concentrated purified fractions was finally confirmed via SDS-PAGE, performed under non-reducing conditions. The fractions corresponding to each elution peak were pooled together and concentrated 2.6×, except in the case of the 910-30 HCLC pool, which was concentrated 4×. Molecular mass marker bands are shown in kDa.

In order to study the effect of the LC and HC gene positioning in a bicistronic vector containing an EMCV IRES element, two anti-SARS-CoV-2 IgG1 were used as model mAbs. Although stable expression in mammalian cells is desired for large-scale production of mAbs, transient expression is often a common means to generate sufficient material for early development and characterization of a recombinant product [31]. As CHO cell lines are most preferred for mAb production, we chose to perform transient transfections in the commercial ExpiCHO™ cell system, as this expression platform allows rapid production of the protein of interest [32]. Figure 2A,B show the viable cell density (VCD) and cell viability of the transfected ExpiCHO™ cells for both LBL-01 and 910-30 mAbs, and for both construct types (LCHC and HCLC). Cells were kept in culture until cell viability decreased to approximately 80%, which occurred at 11 days post-transfection (dpt) for LBL-01 mAb and at 9 dpt for 910-30 mAb. This viability decline is probably due to cytotoxicity of the DNA and the lipid reagent used for transfection and is very similar to the viability decay reported by Jain et al. [32]. According to the spot blot assay of supernatant samples shown in Figure 2C, a higher level of secreted mAbs was found when cells were transfected with the LCHC constructs that carry the LC gene as the first cistron. When the HC gene was placed upstream of the IRES element, mAb production in the supernatant seemed to occur more slowly. mAbs LBL-01 and 910-30 were subsequently purified by protein A affinity chromatography. The recovered mass in each case is shown in Figure 2D, and the SDS-PAGE profile of purified samples is found in Figure 2E. Confirming the supernatant data shown in Figure 2C, a higher amount of purified protein was observed for both mAbs when the cells were transfected with the LC gene in the first cistron (LCHC vectors). According to Ho et al. [14], IRES driven translation (second cistron) is less efficient than a typical cap-dependent translation (first cistron). Therefore, our results give an indication that excess LC expression has a beneficial effect on mAb production, which is in accordance with previous works that have shown this for different mAb subclasses expressed from different vector configurations. Schlatter et al. [33] showed the positive impact of LC excess for the production of an IgG4, with LC and HC genes encoded either in single-gene vectors or in a dual-gene vector containing independent CMV promoter-based transcription units. Ho et al. [14] have found for three different IgG1 molecules that positioning the LC gene upstream of the IRES element led to a higher product concentration, as well as better product quality, when CHO cells were transfected with tricistronic vectors, both for transient transfections and for stable transfections coupled with gene amplification. Bhoskar et al. [34] compared different cell lines at identical culture conditions and a single cell line under varying conditions and showed that LC excess correlates with high mAb productivity and quality. Besides the impact on mAb expression that we observed due to the relative position of LC and HC genes, Figure 2E shows that the expression level also significantly varies for the two different mAbs, since the primary sequence influences critical steps such as folding, assembly, secretion, and intracellular degradation, as discussed by other authors [35,36,37].

### 3.2. Structure Characterization

**Figure 3:**
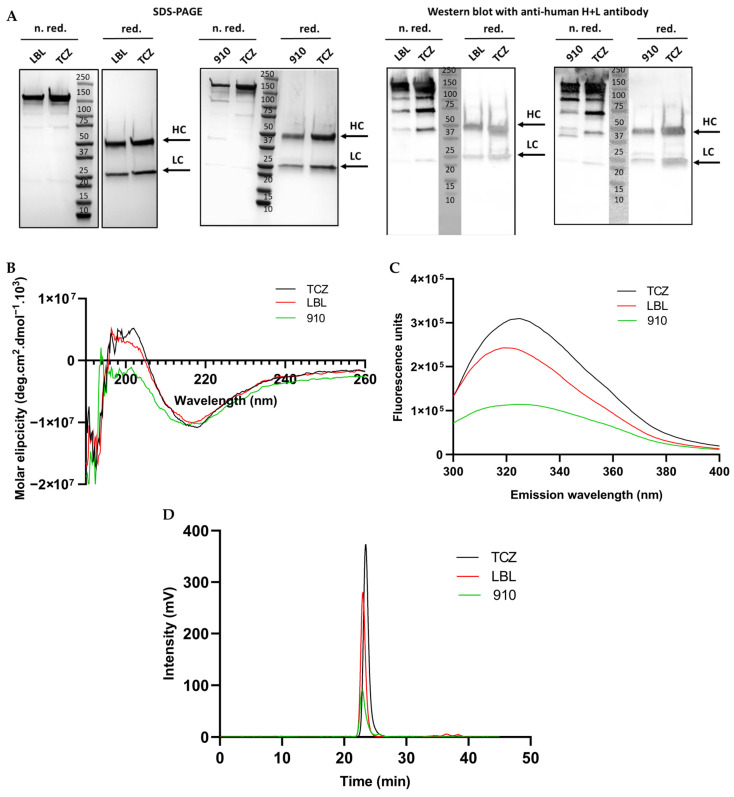
(**A**) SDS-PAGE (**left panel**) and Western blot (**right panel**) of LBL-01, 910-30 and TCZ mAbs for confirmation of purity, identity and molecular mass. Arrows indicate the individual LC and HC bands. Molecular mass marker bands are shown in kDa. (**B**) Circular dichroism (**CD**) spectra obtained for LBL-01, 910-30 and TCZ mAbs. Values of molar ellipticity are represented as the mean of five successive collected replicates. (**C**) Intrinsic fluorescence spectra of tryptophan residues, excited at 280 nm. (**D**) Quaternary structure analysis performed by SEC-HPLC (the chromatograms shown are representative for the experimental duplicates). The commercial therapeutic humanized mAb tocilizumab (TCZ) was included as a reference IgG1 control. Red: reducing conditions. N. red: non-reducing conditions.

Aiming at further characterizing the mAbs, new batches of purified mAbs were analyzed for their molecular mass and purity by SDS-PAGE and Western blot, both under reduced and non-reducing conditions. A commercially available humanized anti-IL6 IgG1 for therapeutic use, Actemra^®^ (tocilizumab, TCZ), was used as an IgG1 control. As shown in Figure 3A, the molecular masses met the expected values of 150, 50 and 25 kDa for IgG1, HC and LC, respectively.

LBL-01 and 910-30 mAbs showed a band profile similar to that of the commercial therapeutic product. The presence of multiple bands under non-reducing conditions for all mAbs (including TCZ), observed both in Figure 2E and Figure 3A, could arise from two phenomena. During protein biosynthesis, unbalanced LC and HC intracellular ratios could lead to mispaired or incompletely folded species, since LC:HC ratio affects both mAb folding and assembly [14]. The second phenomenon could be related to antibody fragmentation artefacts arising from disulfide bond scrambling that occurs due to high-temperature denaturing conditions during sample preparation in non-reducing SDS-PAGE [38,39,40]. Since all bands observed under non-reducing conditions were detected by the anti-human H+L antibody in the Western blots shown in Figure 3A, this indicates a high purity of the mAbs.

As a part of a more detailed characterization of the recombinant mAbs, it is important to assess their primary, secondary, tertiary and quaternary structures [41]. The identity of each purified mAb was confirmed by LC-MS (peptide mapping), with more than 80 and 90% of amino acid sequence coverage of the LC and HC, respectively. Circular dichroism (CD) spectroscopy was employed to analyze the secondary structure of the antibodies (Figure 3B). The far-UV CD spectrum (190–260 nm) provided information about the overall folding pattern of the proteins. Characteristic features observed included a strong negative band around 218 nm, indicative of β-sheet-rich structures, which are typical for immunoglobulins. A secondary peak around 208 nm was also detected, corresponding to α-helical content, although in lower proportions. The spectrum confirmed the antibody’s well-folded conformation, with predominant β-sheet structures and minimal α-helical content, consistent with its expected fold. This structural data supports the proper folding and stability of the antibodies under the experimental conditions [42].

The tertiary structure was qualitatively assessed by measuring the intrinsic fluorescence of tryptophan residues at 280 nm (Figure 3C). For all molecules (commercial tocilizumab, LBL-01 and 910-30) the maximum emission peak was observed at 320–330 nm, corresponding to a class I tryptophan emission spectrum, suggesting that these residues are in a relatively polar and rigid environment [43]. The quaternary structure was further evaluated by SEC-HPLC (Figure 3D), confirming the presence of a uniform peak, at an elution time of 23 min, which is compatible with the IgG1 mass. The highly monodisperse profile, with the main peak accounting for 95–100% of the total integrated area, indicates a high purity of the monomeric IgGs. Remarkably, no apparent high molecular mass species (HMMS) were detected. The presence of HMMS is a concern in biotherapeutics, because they can affect the biological activity of the mAb product and can potentially trigger adverse immune responses to the therapeutic agent, thus having a potential negative impact on safety and efficacy [44].

### 3.3. N-Glycosylation Profile Assessment

**Figure 4:**
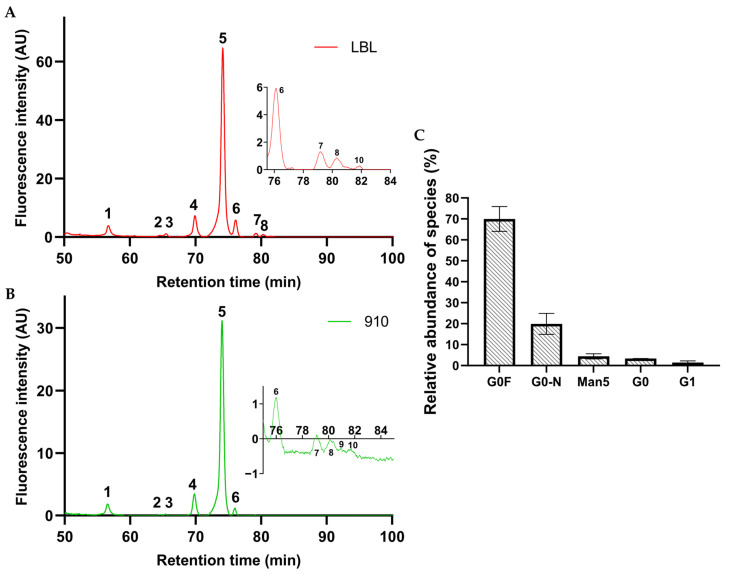
Representative chromatograms of HILIC-HPLC analysis for mAbs (**A**) LBL-01 and (**B**) 910-30, carried out to assess the N-glycan profile. (**C**) LC-MS analysis of mAb LBL-01: relative abundance of glycan species found.

Protein glycosylation influences folding and stability, immunogenicity, efficacy, and serum half-life of glycoproteins, thus giving rise to variability in recombinant glycosylated biotherapeutics. It is also known that glycosylation plays an important role in modulation of Fc interaction with immune effectors [45], which is important in the context of cross-presentation of antigens for T cell activation, antibody-dependent cell-mediated phagocytosis (ADCP), antibody-dependent cellular cytotoxicity (ADCC), and complement-dependent cytotoxicity (CDC) [46]. Particularly, in the case of anti-SARS-CoV-2 mAbs, it was reported that their glycosylation pattern might be associated with different disease outcomes in patients during infection, and also that their intact Fc effector functions could be required for optimal in vivo therapeutic activity [47,48,49]. Consequently, analyzing the glycosylation profile is essential for characterization and quality control of recombinant mAbs [50]. In the present work, we first analyzed both mAbs (LBL-01 and 910-30) by HILIC-HPLC. The analysis involved release of N-glycans with PNGase F followed by derivatization with a fluorophore (2-AB) and separation by HILIC, coupled with fluorescence detection [51]. Similar chromatographic profiles and peak distributions were found for both mAbs, as shown in Figure 4A, B. The possible N-glycan species associated with each peak in Figure 4A,B were assigned according to the GlycoStore Database [52,53] and are listed in Table S1 (Supplementary Materials). From this table, more than one structure could possibly be associated with most of the reported peaks, as similarly described previously by Cruz et al. for another recombinant IgG1 [24].

Additionally, for mAb LBL-01 LC-MS analysis was carried out to enable exact identification of the glycan species, and the percent of relative abundance of the different glycan species was calculated based on the area of the peaks with known *m*/*z* ratios. As shown in Figure 4C, we were able to identify five main glycoforms, which match glycans expected for IgGs produced in CHO cells [54]. The most predominant glycoforms detected were the core fucosylated G0-F, as expected for >90% of recombinant IgGs expressed in CHO cells [55], followed by G0-N, G0, and Man5. Other glycoforms like G1 and G2F, also widely present in IgG1-based products produced in CHO cells [56], were detected in very low proportion (1.4% in the case of G1 and 0.4% in the case of G2F).

### 3.4. Functional Assays

**Figure 5:**
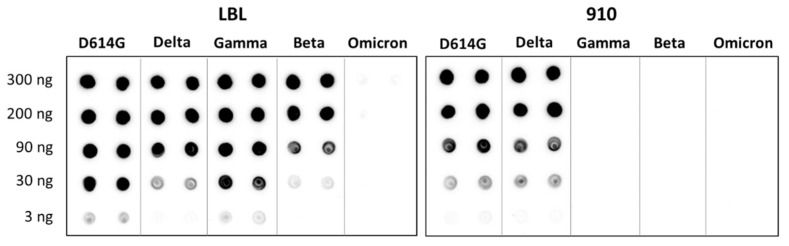
Binding of mAbs LBL-01 (**left**) and 910-30 (**right**) to different recombinant SARS-CoV-2 spike protein variants was evaluated through a spot blot assay.

The functionality of LBL-01 and 910-30 mAbs was studied in terms of their capacities to recognize and bind to recombinant trimeric SARS-CoV-2 spike glycoproteins of different variants. This was first accomplished using a qualitative spot blot assay, in which recombinant spike protein of four different variants (D614G, beta, gamma, delta and omicron BA.2) were first added to a nitrocellulose membrane, to which both LBL-01 and 910-30 mAbs were then added (Figure 5). Further LSPR experiments were conducted to quantitatively determine the affinity of the mAbs for different spike variants. Here the mAbs were immobilized by protein A interaction onto the surface of the LSPR sensors, different concentrations of the target spike proteins were added, and the affinity constant (K_D_) was determined for each case (Table 1). The kinetic curves are shown in Supplementary Materials (Figure S1). Both assays—spot blot and LSPR—showed that mAb LBL-01 was able to target a broader range of recombinant spike variants. As shown in Table 1, no binding was observed to the omicron BA.2 (for both mAbs) and to the gamma variant (only in the case of mAb 910-30). Interestingly, both mAbs were isolated from COVID-19 convalescent patients in 2020, before the emergence of these variants, which emphasizes the importance of the isolating technologies for the identification of potential mAb-based therapeutics for prophylaxis or clinical intervention during pandemic contexts.

It was also described that LBL-01 and 910-30 belong, as other reported neutralizing SARS-CoV-2 mAbs, to a common public clonotype in which the IGHV3-53/3-66 genetic cluster was naturally selected to translate into the HC variable region [26,27]. It is important to note that even though both mAbs are genotypically classified into this group, they notably differ in their binding capacity to the SARS-CoV-2 spike glycoprotein variants, as observed in Figure 5 and in Table 1.

In the absence of somatic hypermutation events, these differences in the diversity of antigenic recognition could be explained in terms of the kappa variable genes usage: in the case of mAb LBL-01 the gene family is the IGKV1_9, whereas in the case of mAb 910-30 it is the IGKV1_33. This is in agreement with Zhang et al. [57], who investigated a broad panel of anti-SARS-CoV-2 mAbs and showed that those mAbs sharing the IGKV1_9 usage presented higher binding affinity to the viral receptor-binding domain (RBD) and a higher neutralization potency, suggesting an enhanced functional efficacy associated with this gene family.

### 3.5. Negative-Staining Images and Reference-Free Class Averages of mAb LBL-01

**Figure 6:**
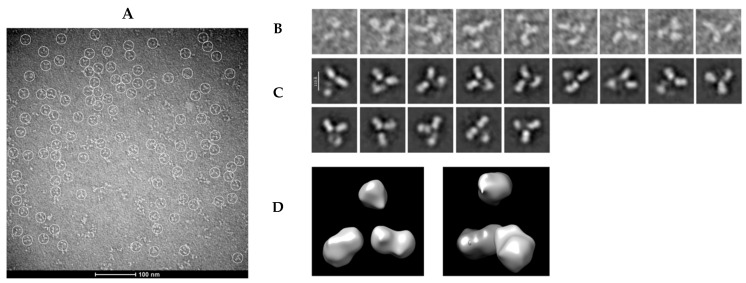
TEM mAb LBL-01 structural characterization. (**A**) Representative negative staining electron microscopy image of mAb LBL-01 particles. White circles highlight individual particles in various orientations. The scale bar represents 100 nm. (**B**) Zoomed-in views of representative extracted particles. (**C**) Selected 2D class averages of mAb LBL-01 particles. (**D**) Negative-stain EM 3D low-resolution reconstruction using CryoSPARC software (version 4.7.1), shown from two different views.

Due to its ability to bind to a broader range of variants, mAb LBL-01 was selected for structural characterization using negative-staining TEM. Images were filtered with a Gaussian low-pass to 5 Å. The survey micrographs revealed evenly distributed particles exhibiting the characteristic “Y”-shaped structure typical of immunoglobulins (circled in Figure 6A). Two-dimensional class averages further confirmed the general “Y” architecture; however, the limited particle count combined with the antibody’s conformational flexibility, resulted in blurred or fuzzy regions in several classes (Figure 6B,C). This variability is consistent with the flexibility observed in other immunoglobulins, particularly at the hinge, where the Fab arms and the Fc stem can adopt different orientations. Such flexibility is a critical property of antiviral neutralizing mAbs, enhancing their ability to search for and effectively engage antigens [58].

A low-resolution 3D reconstruction, generated from 14 high-quality 2D classes (Figure 6D), confirmed the basic architecture of mAb LBL-01. However, due to the limited particle count and inherent molecular flexibility, finer structural details could not be resolved. Although negative-staining TEM data does not typically achieve atomic resolution, the low-resolution map of mAb LBL-01 obtained provides valuable structural insights into this previously uncharacterized antibody. While the current map provides an overview of its architecture, determining the exact arrangement and functional states of the Fab and Fc domains will require single-particle cryo-electron microscopy (cryo-EM). Nonetheless, this preliminary dataset establishes a crucial foundation for more detailed structural investigations.

## 4. Conclusions

In this study, two anti-SARS-CoV-2 mAbs were expressed in CHO cells and underwent structural and functional characterization. Regarding mAb expression, bicistronic vectors were constructed for both mAbs (LBL-01 and 910-30), and it was observed that higher expression levels were obtained when the gene coding for the light chain was positioned as the first cistron. Both mAbs were then produced in higher amount and purified by protein-A chromatography. Analyses of secondary, tertiary and quaternary structure, as well as N-glycosylation pattern, revealed similar quality attributes for both mAbs. However, although both antibodies belong to the IGHV3-53/3-66 gene usage group, both blotting and LSPR techniques showed that LBL-01 mAb has a broader ability to bind the spike protein of different SARS-CoV-2 variants. High-quality 2D class averages of mAb LBL-01 were obtained by negative staining transmission electron microscopy and confirmed the basic mAb architecture. The preliminary TEM dataset obtained did not achieve, as expected, atomic resolution, but allowed for a low-resolution 3D reconstruction to be generated and established a crucial foundation for the future determination of the functional states of Fab and Fc domains by cryo-electron microscopy.

## Figures and Tables

**Table 1:** Affinity parameters found by LSPR for mAbs LBL-01 and 910-30 binding to different spike protein variants.

	LBL-01			910-30		
Variant	*K_on_* (M^−1^ s^−1^)	*K_off_* (s^−1^)	*K_D_* (M)	*K_on_* (M^−1^ s^−1^)	*K_off_* (s^−1^)	*K_D_* (M)
D614G	8.63 ± 0.00 × 10^4^	7.21 ± 0.45 × 10^−^^5^	8.36 ± 0.05 × 10^−^^10^	9.06 ± 0.02 × 10^4^	4.05 ± 0.78 × 10^−^^5^	4.47 ± 0.86 × 10^−^^10^
Delta	1.17 ± 0.00 × 10^5^	2.30 ± 0.00 × 10^−^^4^	1.96 ± 0.03 × 10^−^^9^	1.43 ± 0.04 × 10^5^	2.56 ± 0.01 × 10^−^^3^	1.79 ± 0.01 × 10^−^^8^
Gamma	8.38 ± 0.01 × 10^4^	6.41 ± 0.56 × 10^−^^5^	7.66 ± 0.67 × 10^−^^10^	ND	ND	ND
Omicron	ND	ND	ND	ND	ND	ND

ND: Not Detectable.

## Data Availability

The original contributions presented in this study are included in the article/Supplementary Materials. The data and mate-rials are available from the corresponding author upon reasonable request. Further inquiries can be directed to the corre-sponding author.

## Supplementary Materials

The following supporting information can be downloaded at https://www.mdpi.com/article/10.3390/antib14040086/s1: Figure S1: LSPR sensorgrams of mAbs (A–C) LBL-01 and (D and E) 910-30.; Table S1. Possible N-glycans found by HILIC-HPLC in LBL-01 and 910-30 mAbs.
