# Supplementary material for: Structural and Functional Characterization of Anti-SARS-CoV-2 Spike Monoclonal Antibodies Produced via Bicistronic Expression in CHO Cells
Source: Antibodies (Basel). 2025 Oct 9;14(4):86. doi: 10.3390/antib14040086 (PMC12550980; doi:10.3390/antib14040086)
Supplement: Supplementary file 1 [file antibodies-14-00086-s001.zip › antibodies-3798070-supplementary.pdf]

Supplementary Materials

Table S1. Possible N-glycans found by HILIC-HPLC in LBL-01 and 910-30 mAbs. The retention times (RTs) were converted to glucose units (GU) based on the injection of a hydrolyzed glucose ladder. The main peaks were assigned according to the GlycoStore Database. The standard deviation was calculated from two analytical replicates.

	RT (min)		GU			
Peak	LBL-01	910-30	LBL-01	910-30	Possible structures (Oxford notation)	Short name used with IgG glycans
1	56.7 ± 0.1	56.1 ± 0.1	3.3 ± 0.0	3.3 ± 0.0	M1; M2	Fragment of Man3
2	64.5 ± 0.1	64.5 ± 0.0	4.0 ± 0.0	4.0 ± 0.0	F(6)M2	Fragment of Man3F
3	65.5 ± 0.1	65.4 ± 0.0	4.1 ± 0.0	4.1 ± 0.0	F(3)M2	Fragment of Man3F
4	69.9 ± 0.0	69.7 ± 0.1	4.5 ± 0.0	4.5 ± 0.0	M3; F(3)F(6)M2	Man3; G0F2
5	74.1 ± 0.0	74.0 ± 0.1	5.0 ± 0.0	5.0 ± 0.0	F(6)M3; A1[6]; M3B; A1; A1[3]; M2A1G(4)1	Man3F; G0-N[6]; Man3B; G0-N; G0-N[3]; Fragment of G1
6	76.1 ± 0.1	76.0 ± 0.1	5.2 ± 0.0	5.2 ± 0.0	A1B; M4; F(3)M3; A1[6]B; A1[3]B	G0B-N; Man4; Man3F; G0B-N[6]; G0B-N[3]
7	79.2 ± 0.0	79.0 ± 0.1	5.6 ± 0.0	5.6 ± 0.1	F(6)A1; F(6)M2A1[3]G(4)1; F(6)M2A1[3]G(4)1S1	G0-FN; Fragment of G1F-N; Fragment of G1FS-N
8	80.3 ± 0.0	80.1 ± 0.0	5.7 ± 0.0	5.7 ± 0.0	A1G1; F(6)M4; F(6)F(3)M3; A2B	G1-N; Man(4)F; G0F2; G0B
9	-	81.1 ± 0.3	-	5.8 ± 0.0	A1[6]G(4)1; A1[3]G(4)1	G1F-N[6]; G1F-N[3]
10	81.8 ± 0.1	81.7 ± 0.0	5.9 ± 0.0	5.9 ± 0.0	A3; F(6)A2	G0+N(tri); G0F

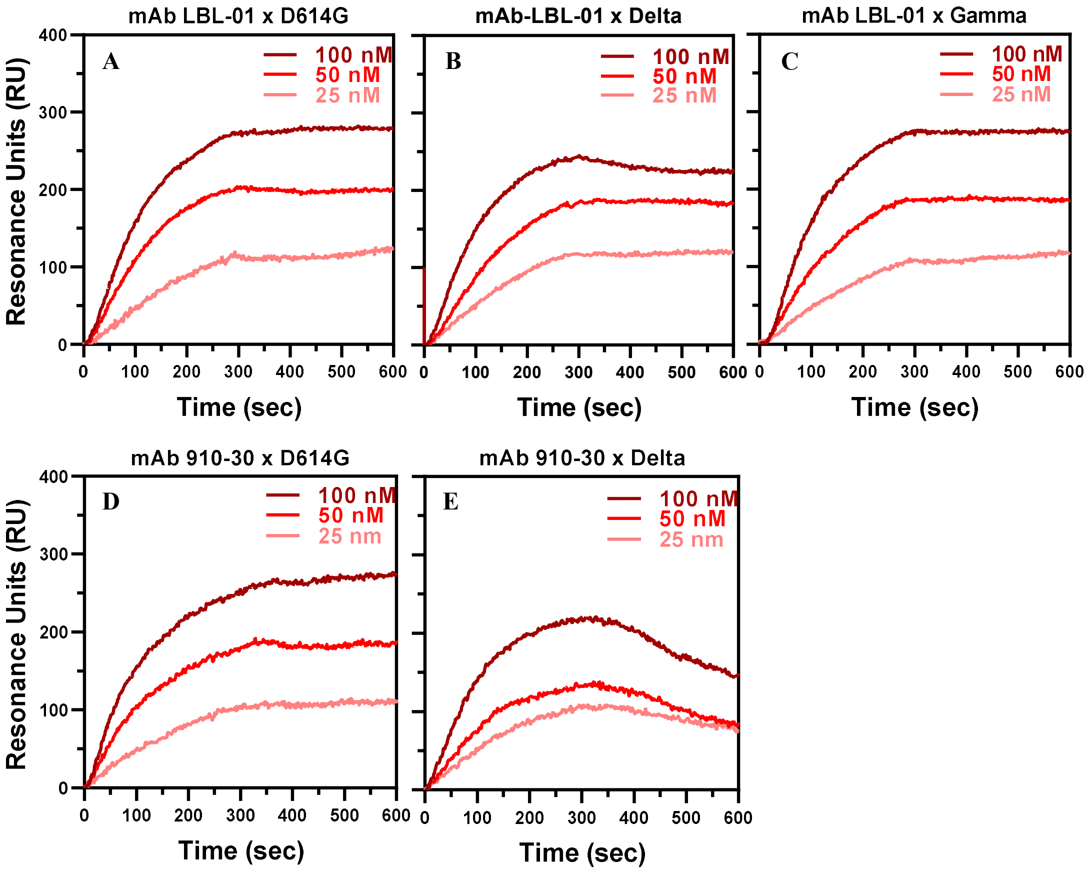

Figure S1. LSPR sensorgrams of mAbs (A–C) LBL-01 and (D and E) 910-30. The interactions between the mAbs immobilized on protein A sensors and spike glycoprotein corresponding to D614G, delta and gamma variants were measured at different analyte concentrations using LSPR. The signals shown correspond to the averages of two analytical replicates.
